# How to use *DIALS* to process chemical crystallography 3D ED rotation data from pixel array detectors

**DOI:** 10.1107/S2053229624011148

**Published:** 2025-01-01

**Authors:** Angelina Vypritskaia, Xiaodong Zou, Taimin Yang, David Geoffrey Waterman

**Affiliations:** a Department of Materials and Environmental Chemistry, Stockholm University, Stockholm SE-106, Sweden; b Research Complex at Harwell UKRI–STFC Rutherford Appleton Laboratory Harwell Didcot Oxfordshire OX11 0FA United Kingdom; University of Delaware, USA

**Keywords:** 3D ED, MicroED, DIALS, chemical crystallography, crystal structure

## Abstract

The *DIALS* package provides a set of tools for crystallographic data processing. The open-source nature of the project, and a flexible inter­face in which individual command-line pro­grams each have a dedicated job, have enabled the adaptation of *DIALS* to a wide range of experiment types, including electron diffraction. Here we present detailed instructions for the use of *DIALS* to process chemical crystallography diffraction data from con­tin­u­ous rotation electron diffraction experiments. We demonstrate processing and structure solution from three different samples from three different instruments, including two commercial instruments dedicated to electron diffraction. Each instrument has a pixel array detector, allowing low-noise data to be obtained, resulting in high quality structures. Various new features were added to *DIALS* to simplify the workflow for these use cases. These are described in detail, along with useful pro­gram options for electron diffraction work.

## Abstract 2

Three examples of data processing of chemical crystallography 3D ED rotation data using the *DIALS* package are presented. These examples are taken from a standard transmission electron microscope, and from two commercial instruments, each equipped with a low-noise pixel array detector optimized for diffraction studies.

## Introduction

Electron crystallography has rapidly evolved over the past decade due to the development of 3D acquisition and electron diffraction data analysis protocols (Gemmi *et al.*, 2019 ▸). These advancements enable us to study the structure of materials with enhanced data quality and acquisition speed, which has been proved by the successful determination of various structures from minerals (Gemmi *et al.*, 2019 ▸), small organic mol­ecules (Andrusenko & Gemmi, 2022 ▸), zeolites (Cho *et al.*, 2023 ▸) and metal–organic frameworks (Samperisi *et al.*, 2022 ▸; Huang *et al.*, 2021 ▸) to proteins (Clabbers & Xu, 2021 ▸).

Software development has also been crucial in supporting these new techniques, by providing robust data processing. *DIALS* (Winter *et al.*, 2018 ▸), originally developed for X-ray crystallography data processing, was later extended to work with electron diffraction data (Clabbers *et al.*, 2018 ▸), with an aim to provide unified software for crystallographic data processing. *DIALS* is freely available for download from https://dials.github.io/ under the terms of the BSD-3 license. Linux and Mac packages are provided. Additionally, *CCP4* (Agirre *et al.*, 2023 ▸) and *conda-forge* provide builds for Windows. The *DIALS* package consists of a suite of com­mand-line pro­grams, each of which performs a discrete step of data processing. In X-ray crystallography, where comprehensive metadata are usually associated with the image data, data processing may be achieved with simple commands that mainly use pro­gram defaults (the following is based on the X-ray small-mol­ecule tutorial at the *DIALS* website):

dials.import ../data/*.cbf

dials.find_spots imported.expt

dials.index imported.expt strong.refl\

space_group=P222

dials.refine indexed.expt indexed.refl

dials.integrate refined.expt refined.refl

dials.symmetry integrated.expt integrated.refl

dials.scale symmetrized.expt symmetrized.refl

dials.export scaled.expt scaled.refl\

format=shelx composition=CHNOS

In our research, we examine the processing of 3D electron diffraction (3D ED) data collected in three different setups: con­tin­u­ous rotational electron diffraction with calibration images (cRED) collected on an electron microscope at Stockholm University, data collected on a Rigaku Synergy-ED instrument at the UK National Electron Diffraction Facility and data from an Eldico ED-1 dif­frac­tom­eter at the Eldico Customer Experience Center in Basel. Each configuration has its unique benefits and challenges for collecting and analyzing data. We describe how *DIALS* is applied and adapted for each experimental scenario. We have included additional functionalities in *DIALS* according to the requirements encountered during our investigations. The new features have made the data processing workflow more efficient while maintaining *DIALS* as a reliable tool for electron crystallography analysis. The examples are based on publicly-available data, and we provide example *DIALS* processing scripts with detailed explanations of pro­gram parameters. This enables users to follow along and reproduce the results shown in the article. All data processing results shown were produced with *DIALS* Version 3.21.1, which contains the new features introduced to improve the handling of electron diffraction data.

## cRED data

The development of fast read-out electron detectors, such as Timepix (van Genderen *et al.*, 2016 ▸), made it possible to collect electron diffraction data con­tin­u­ously, leading to the development of con­tin­u­ous rotational electron diffraction (cRED) (Cichocka *et al.*, 2018 ▸), also known as MicroED (Shi *et al.*, 2013 ▸). In a cRED experiment, the goniometer con­tin­u­ously rotates at a constant speed while the diffraction patterns are recorded over a certain exposure time. cRED allows faster data collection and more complete sampling of reciprocal space than stepwise tilt data collection, making it particularly useful for investigating radiation-sensitive materials, and it has been used successfully for studying various inorganic and organic samples and proteins, including a number of zeolites, MOFs and pharmaceuticals. The only hardware requirement for cRED data collection is a transmission electron microscope with a suitably high-quality single tilt sample holder and a camera. Merging several 3D ED data sets allows completeness to be increased (Ge *et al.*, 2021 ▸) and overall data quality to be improved (Xu *et al.*, 2018 ▸).

### Electron nanocrystallography (NanED) Round Robin samples

NanED (https://naned.eu/) is a collaborative training pro­gram funded by the EU, aimed at training a new generation of electron crystallographers. This is intended to make it easier for electron diffraction methods to become widely accepted and used across academic and industrial environments.

Within the framework of the NanED project, the Round Robin deliverable was set up with two main goals: to train new researchers in 3D ED techniques using well-known samples and to compare results across different labs using a variety of microscopes and analysis methods (Gemmi *et al.*, 2023 ▸). As an example, we selected one of the Round Robin samples, natrolite, a mineral from a zeolite family, containing vertex-sharing *T*O_4_ tetra­hedra (*T* = Si or Al).

Crystals of the natrolite sample were crushed in an agate mortar. The powder was dispersed in ethanol and ultrasonicated for 3 min. A drop of the suspension was placed on a copper grid with lacey carbon film. Data collection was performed at room temperature.

cRED data of the natrolite crystals were collected on a JEM-2100 LaB_6_ microscope, with an accelerating voltage of 200 kV. A Timepix hybrid pixel detector and the *Instamatic* software (Cichocka *et al.*, 2018 ▸) were used for data collection. The resulting data sets consist of SMV format images. During data collection using *Instamatic*, every *n*th diffraction pattern is defocused (usually *n* equals 10 or 20) to track the crystal during rotation. The beam diameter was around 700 nm, and if the crystal was seen to move away from the beam centre this was adjusted by movement of the sample stage. This prevents the loss of the crystal due to crystal movement and allows data collection with large rotation ranges (Cichocka *et al.*, 2018 ▸). For the four data sets investigated here, the tilt per image ranged from 0.2296 to 0.2318°, and the total tilt ranged from 90.24 to 118.91°. Beam centre drift of a few pixels was evident over the course of data collection. However, diffraction patterns were not centred as the drift was tolerated in data processing.

### Data processing

The *DIALS* commands used to integrate the natrolite data sets were similar in each case. For illustration, commands for processing a single natrolite data set within a *BASH* shell on a Linux computer are reproduced here.

dials.import "$NATROLITE_DATA"/Data1/SMV/data/*.img\

geometry.goniometer.axis=-0.6204,-0.7843,0.0000\

panel.gain=2.9

dials.generate_mask imported.expt\

untrusted.rectangle=0,516,255,261\

untrusted.rectangle=255,261,0,516

dials.apply_mask imported.expt mask=pixels.mask

dials.find_spots masked.expt\

exclude_images_multiple=20\

d_max=10 d_min=0.6 gain=0.5

dials.index masked.expt strong.refl\

detector.fix=distance space_group=F222

dials.reindex indexed.expt indexed.refl\

change_of_basis_op=b,c,a space_group=Fdd2

dials.refine reindexed.expt reindexed.refl\

detector.fix=distance

dials.integrate refined.expt refined.refl\

prediction.d_min=0.6\

exclude_images_multiple=20

This assumes that the path to the parent directory containing the natrolite data sets is given by the variable NATROLITE_DATA. Key points regarding the behaviour of *DIALS* during the execution of these commands are explained in the following sections.

#### Determining the rotation axis

The SMV format has no robust metadata standard and diffraction geometry metadata is typically incomplete (Waterman *et al.*, 2023 ▸). While the beam centre is written to the image header by *Instamatic*, and is read by *DIALS*, there is no header entry to represent the orientation of the rotation axis in the image. The *dxtbx* format class (Parkhurst *et al.*, 2014 ▸) used to read the SMV images from this microscope assumes a default orientation, which was determined some years ago. During processing of the Round Robin data sets it was discovered that the rotation axis now differs slightly from this default.

An algorithm for determining the rotation axis orientation from diffraction data has been reported by Kolb *et al.* (2009 ▸), and since then has been adopted by other packages, such as *PETS2* (Palatinus *et al.*, 2019 ▸) and *edtools* (Cichocka *et al.*, 2018 ▸). The pro­gram *dials.find_rotation_axis* also implements this algorithm and is a direct adaptation of the open source *edtools* version. The algorithm is effective in the case of good quality spot-finding results from a single crystal. If a large number of noise peaks are found as spots, then the algorithm may fail to identify the correct orientation. We ran *dials.find_rotation_axis* on several of the Round Robin data sets, then set the same consensus value *via* the geometry.goniometer.axis= option of *dials.import* for all further processing. For any one data set, the *dials.find_rotation_axis* command was run after spot-finding as follows:

dials.find_rotation_axis masked.expt strong.refl

#### Determining the detector gain

The Timepix detector is able to count events with energy higher than a user-defined threshold (van Genderen *et al.*, 2016 ▸). Due to scattering of high-energy electrons within the silicon sensor, charge spread beyond the point of incidence may result in counts within neighbouring pixels. The detector gain, defined as the number of detected counts per incident electron, is expected to be greater than 1 due to this charge sharing. The value of the gain is important for spot-finding, and for a realistic scale of the integrated intensity error estimates, σ(*I*), before further modifications to the error model are made in scaling. For a detector without a pedestal offset, the gain can be estimated by comparing the ob­ser­ved index of dispersion with the value 1.0, which is the expected value for a perfect counting detector. The pro­gram *dials.estimate_gain* performs this calculation within a local region of background scatter, but this method is known to underestimate the true gain for a detector with non-negligible point spread (Water­man & Evans, 2010 ▸; Clabbers *et al.*, 2018 ▸).

A reviewer of an earlier version of this article pointed out an alternative way to experimentally measure gain for a PAD. This method is rather direct and subject to fewer assumptions than approaches based on variance of the signal. Illumination of the detector with a very low dose allows identification of single electron impacts, which generally form clusters of a few pixels. In this context, the gain may be known as the ‘event multiplicity’, and is given by the average number of counts across correctly identified single electron impacts (Fernandez-Perez *et al.*, 2021 ▸). We collected low-dose images of 200 keV electrons from the Timepix detector with the software *SoPhy*, provided by Amsterdam Scientific Instruments. The smallest con­denser aperture (10 µm) was used, and the spot size was 5, with magnification at 600 000× and 0.1 s frame exposure. An analysis of event clusters from 40 images resulted in the value 2.9 for the event multiplicity, which we then set at import using the panel.gain= parameter (see Section S1 in the supporting information for further details).

We found that the results from *dials.find_spots* using this higher gain estimate tended to omit weaker but clearly visible spots, as inspected with the *dials.image_viewer*. For this reason, we added the parameter gain=0.5 to the *dials.find_spots* command. Here it acts as a multiplier for the panel gain set at import, so that the gain assumed by the spot-finding algorithm is 1.45 rather than 2.9. This increased the sensitivity of spot-finding, recovering some of the weaker spots. While the panel gain set at import persists throughout the steps of data processing with *DIALS*, the adjustment performed for spot-finding affects that step only.

#### Masking the central cross

The Timepix quad detector consists of four 256 × 256 pixel chips, each of which has an outer border composed of wider pixels (van Genderen *et al.*, 2016 ▸). The area where the chips join forms a central cross in the image of two pixels width, with greater intensity values than the surrounding pixels due to their larger physical size. After conversion by the *Instamatic* software, the overall image size is expanded to 516 × 516 pixels to account for the size of the central cross region. The intensity of pixels within the cross is adjusted to the scale of the surrounding pixels; however, the profiles of reflections recorded in the cross are affected and are not well modelled by reflection profiles recorded elsewhere on the detector. For this reason, for pro­grams like *DIALS* that do integration by profile fitting, it is not recommended to use the pixels in this cross. For the cRED processing presented here, this was achieved by explicitly masking those pixels using the *dials.generate_mask* and *dials.apply_mask* commands. These commands have been left in the script above as an example; however, for simplicity, this mask has now been added to the format class used to read the images.

#### Excluding crystal tracking images

*DIALS* can exclude images from spot-finding, integration and scaling, using a consistent syntax given by exclude_images=exp:start:stop, where exp gives the experiment number, and start and stop give the inclusive range of images to exclude from consideration. Multiple ranges can be provided in a single definition by comma-separated values, and multiple definitions of the parameter will be combined. While flexible, this syntax was found to be inconvenient for scripting the processing of cRED data. To simplify the inter­face, we added the parameter exclude_images_multiple=n, where, for example, *n* = 20. This is automatically expanded into the appropriate image exclusion definition to regularly exclude each image number exactly divisible by *n* within the image range of the data set. The collection of crystal tracking images is a feature provided by *Instamatic*, so the new parameter makes it easier to work with *DIALS* on data collected by *Instamatic* on any instrument it supports.

Image exclusions defined in spot-finding act as a mask, setting all pixels to ‘invalid’ on the excluded images. Diffraction spots that are inter­rupted by a tracking image may be found within two separate spot shoeboxes (before and after the tracking image). Typically, *dials.index* will only index one of the two in these cases, leading to error in the centroid position and profile for that spot. Nevertheless, these effects are mitigated by the diffraction geometry refinement and profile modelling using data from the whole scan.

**Figure 1:**
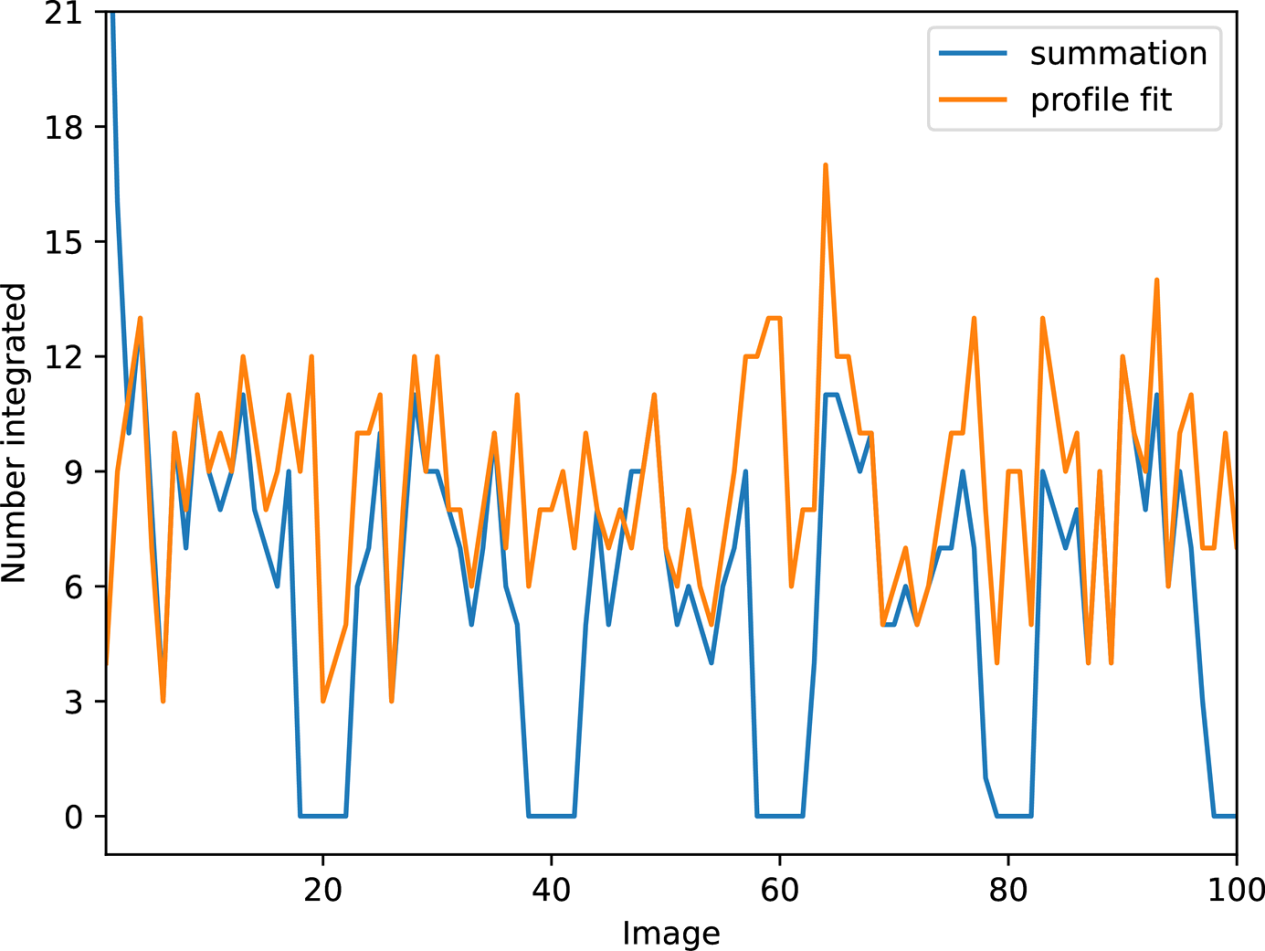
Comparison of the number of reflections integrated by either summation integration or profile fitting on each of the first 100 images of the first natrolite data set (Data1). Calibration image numbers are located at each multiple of 20.

During integration, image exclusion also acts like a mask. For spots whose peak region is inter­sected by a tracking image, no summation integration intensity estimate is possible. There­fore, a plot of the number of summation integrated spots shows wide gaps around the calibration images, as shown by Fig. 1 ▸. Profile fitting is able to recover an intensity estimate even when the peak region contains some invalid pixels. This is controlled by the valid_foreground_threshold parameter for *dials.integrate*, which is set to 0.75 by default. Thus, as long as 75% of the pixels forming the diffraction spot are outside the excluded image, or not otherwise masked, then a profile fitting estimate will be recorded. Fig. 1 ▸ shows that the number of profile-fitted intensities drops at the tracking images, but not to zero. The overall number of profile-fitted intensities is about 50% higher than the number of summation integrated intensities for this data set.

This procedure was seen to work well for data sets we tried where every 20th image was a tracking image. In some cases where *n* = 10 instead we saw a failure in profile fitting because *dials.integrate* could not create a profile model. This occurred when the full extent of the ob­ser­ved rocking curve of strong reflections exceeded the spacing between tracking images. Thus, the spacing between these images should be chosen carefully based on the properties of the sample.

#### Indexing and refinement

The default 3D FFT indexing method of *DIALS* usually works well with these cRED data sets. Indexing success hinges on the quality of spot centroid data, and noise peaks may cause problems with basis vector determination. Pixel array detectors allow data collection without a beamstop and, unless there is an energy filter, this reveals a cone of significant background at low angle around the beam centre, caused by inelastic scatter of the direct beam. This typically leads to a spherical region around the centre of reciprocal space within which many noise peaks are found. The simplest way to avoid this and provide better data for indexing is to set a low resolution limit in Å, such as d_max=10, for *dials.find_spots*.

**Figure 2:**
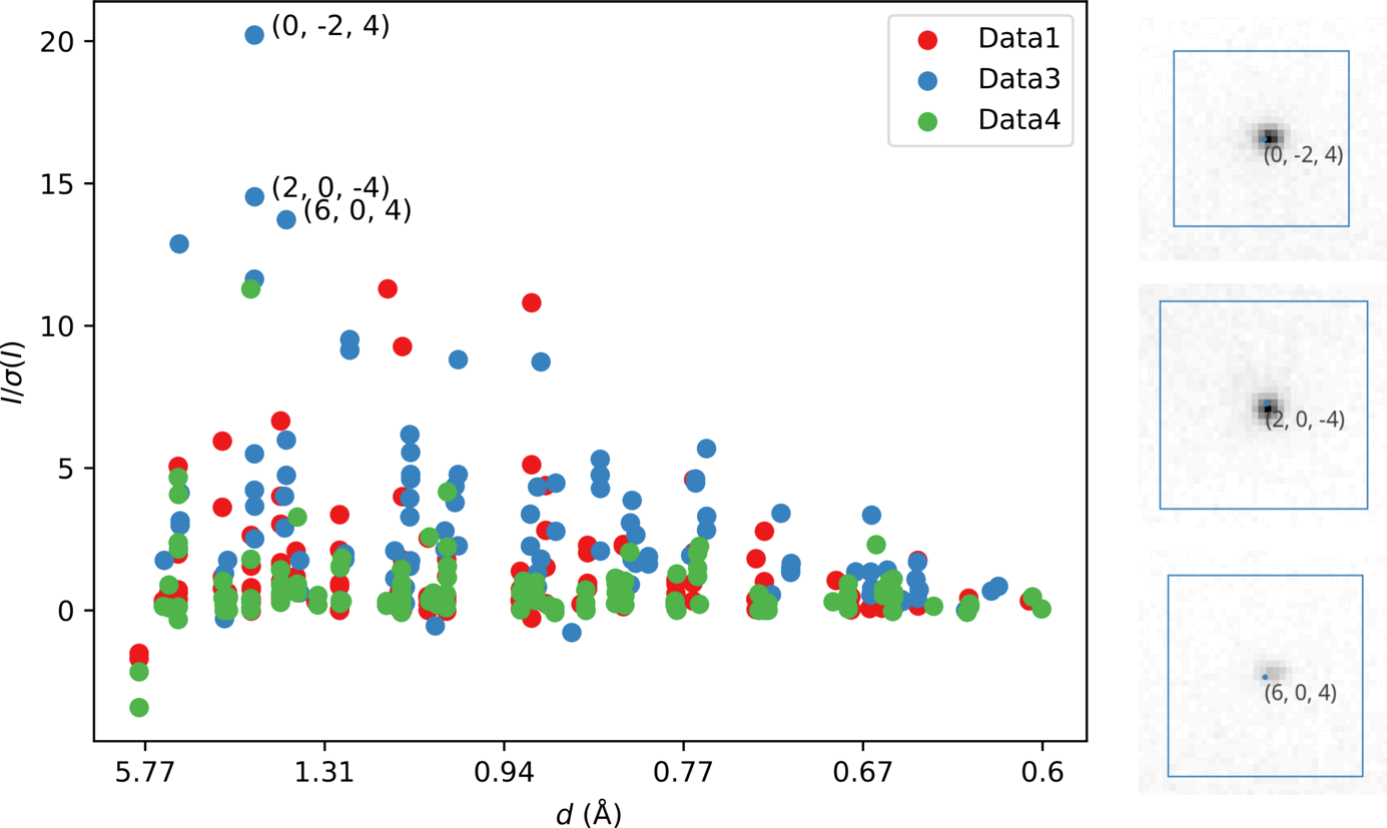
*I*/σ(*I*) *versus* resolution for 364 reflections from the scaled natrolite data sets that should be systematically absent in the space group *Fdd*2 under the kinematic approximation. The points are coloured according to which of the three crystals they come from. A few reflections from each crystal show significant violation of their expected absence. On the right, images from the *dials.image_viewer* of the three spots with highest *I*/σ(*I*) are shown, clearly indicating that these violations are present in the data and are not an artefact of data processing.

Unless a space group is provided, *dials.index* will return a triclinic solution. In this case, the space group for natrolite is known to be *Fdd*2 (Fig. 2 ▸). However, *dials.index* will return an ortho­rhom­bic solution by convention with *a* < *b* < *c*, whereas the correct solution has *c* < *a* < *b*. Rather than setting the space group to *Fdd*2 immediately, we first specified the subgroup *F*222 to ensure that the unit-cell refinement performed by *dials.index* was appropriately constrained. Once this solution was written out, the pro­gram *dials.reindex* was used to change the basis and to set the correct space group.

In some cases with higher symmetry space groups, enforcing lattice constraints can lead to excessively high root-mean-square deviations between predicted and ob­ser­ved spot positions, negatively affecting integrated data quality. This can occur if unmodelled distortions from post-sample lenses (Brázda *et al.*, 2022 ▸), or other deviations from the refined diffraction geometry, are present. In such cases, it can be useful to process data using the triclinic solution, in which the unmodelled geometry errors may be transferred, to some extent, to errors in the unit-cell parameters. After integration, automatic determination of symmetry may be performed using tools such as *dials.symmetry* or *dials.cosym* (Gildea & Winter, 2018 ▸), to ensure scaling is performed with the correct Laue class. In the experience of the authors, however, much care must be taken when using the *DIALS* symmetry-determination tools with 3D ED data. The generally higher errors on intensities due to effects such as multiple scattering in such data as compared to X-ray data can lead to incorrect results.

Simultaneous refinement of the detector distance and the unit-cell parameters is not usually possible for 3D ED data unless a restraint is applied (Clabbers *et al.*, 2018 ▸). Here we simply fixed the detector distance using the detector.fix=distance parameter, for the *dials.index* and *dials.refine* commands, both of which perform geometry refinement. This implies that errors in the calibrated effective detector distance will be expressed as errors in the refined unit-cell lengths. It may be possible to correct such errors after structure solution during model refinement (Gruene *et al.*, 2022 ▸).

After refinement of a static model for the experiment, *dials.refine* performs a scan-varying refinement, in which parameters are allowed to vary as a smoothed function of position within the rotation scan (Waterman *et al.*, 2016 ▸; Clabbers *et al.*, 2018 ▸). The default behaviour is to allow the crystal orientation and unit-cell parameters to vary in this way, while other parameters, such as the beam direction, and the detector position and orientation are refined to static values. In the case of 3D ED data collection from nanocrystals using a selected area aperture, the beam is generally larger than the sample, ensuring there is no apparent unit-cell variation due to the sampling of different mosaic blocks during data collection. Changes to the unit-cell values due to radiation damage may still be present, but for the natrolite data these are expected to be small. Other errors, such as drift of the direct beam and distortion in the diffraction patterns caused by post-sample lenses, were not directly accounted for here; however, their effects may be partially compensated for by the scan-varying refinement of crystal parameters. There was a small im­provement in merging statistics seen by allowing the unit cell to vary smoothly, so we used this model for further processing.

#### Determining the best overall unit cell

The unit cells determined by *dials.refine* are based on refinement against the indexed strong spot centroids, without background subtraction. A more precise unit-cell refinement can be performed after integration using the recalculated integrated spot centroids, including background subtraction. The pro­gram *dials.two_theta_refine* performs this analysis. By using a target function based on the reflection 2θ scattering angles rather than the position of reflection impacts, the data from multiple crystals at different orientations are combined to produce a single best (in a least-squares sense) cell for all data sets. The command used to do this for a combination of three of the natrolite data sets was

dials.two_theta_refine\

"$NATROLITE_PROC"/Data1/integrated.{expt,refl}\

"$NATROLITE_PROC"/Data3/integrated.{expt,refl}\

"$NATROLITE_PROC"/Data4/integrated.{expt,refl}

This assumes that the processing of each data set was performed in its own directory, under a common parent directory, the path to which is stored in the variable NATROLITE_PROC. The command produced the file refined_cell.expt, in which the single unit cell with standard uncertainties was given as *a* = 18.640 (9), *b* = 18.788 (4) and *c* = 6.8419 (16) Å.

#### Scaling multiple data sets

Combining multiple data sets may be important in cRED in order to increase completeness and to help average out the typically high errors (compared to X-ray crystallography) (Xu *et al.*, 2018 ▸; Ge *et al.*, 2021 ▸). As long as the integrated data sets are consistently indexed, then they can be jointly-scaled by *dials.scale* (Beilsten-Edmands *et al.*, 2020 ▸). First we tried joint scaling of all four natrolite data sets. Inspection of the plot of scale factor against batch reported in the dials.scale.html file indicated that the second data set, Data2, comprised considerably weaker diffraction intensities than the other data sets. Exclusion of this data set produced better merging statistics and a lower refinement *R*_1_ value. Therefore, we discarded that data set and scaled the three remaining natrolite data sets using the command given here, run in the same directory as the refined_cell.expt file produced by *dials.two_theta_refine*.

dials.scale refined_cell.expt\

"$NATROLITE_PROC"/Data1/integrated.refl\

"$NATROLITE_PROC"/Data3/integrated.refl\

"$NATROLITE_PROC"/Data4/integrated.refl\

merging.nbins=10\

d_min=0.61

dials.export scaled.expt scaled.refl\

format=shelx\

composition="Si Al Na O H"

The first option passed to *dials.scale* after the sequence of integrated data sets is merging.nbins=10. This option does not change the behaviour of the scaling algorithm, but just sets the number of bins used for reporting merging statistics. The default value is 20, but for small unit cells this can lead to noisy values for the merging statistics, as there are relatively few reflections in each bin. We found that reducing the value to 10 produced more reasonable values for merging statistics in the inner and outer resolution shells, reported in tables such as Table 1 ▸.

The second option passed to *dials.scale*, d_min=0.61, sets the high resolution limit for reflections included in scaling and merging.

### Structure solution and refinement

After scaling, the unmerged data were exported using *dials.export*, specifying the composition to produce dials.ins and dials.hkl files suitable for immediate use by *SHELXT* (Sheldrick, 2015*a* ▸). Structure solution was straightforward with the data processed as described; however, it is worth raising a note of caution. Prior to estimating the detector gain using the method described in Section 2.2.2, we used a lower value for the detector gain, and in this case found that the structure could be solved by *SHELXT* Version 2014/5, but Version 2018/2 failed to produce a solution. A feature introduced in Version 2018/2 is the rejection of space groups where systematic absences have a mean *I*/σ(*I*) > 5 (as noted at https://shelx.uni-goettingen.de/changes.php). *DIALS* always integrates reflections that should be absent due to screw axes or glide planes given the supposed space group, and we suspected that systematic absence violation was the cause of the failure to solve the structure in this case.

Once the gain was better estimated by the event multiplicity method, the structure was solved by either version of *SHELXT*. Nevertheless, an analysis of the systematic absences shown in Fig. 2 ▸ revealed numerous reflections that are expected to be absent in fact still had significant diffraction intensity. These violations of the reflection conditions are expected in 3D ED due to multiple scattering. Although increasing the gain value, which led to an increase in the sigma estimates for reflections, was enough to avoid failure in *SHELXT* in this case, it is worth being aware of this issue, particularly in cases where crystals are relatively thick, or the sigma estimates on intensities may be severely underestimated. The error model refined by *dials.scale* defaults to a physical model similar to that in *AIMLESS* (Evans & Murshudov, 2013 ▸), which works well for synchrotron X-ray data sets. In general, we suspect that for 3D ED data sets the error estimates from *DIALS* may be too small. The issue of optimal error modelling for 3D ED data has been addressed by Khouchen *et al.* (2023 ▸). This model has not yet been assessed within *DIALS*.

**Figure 3:**
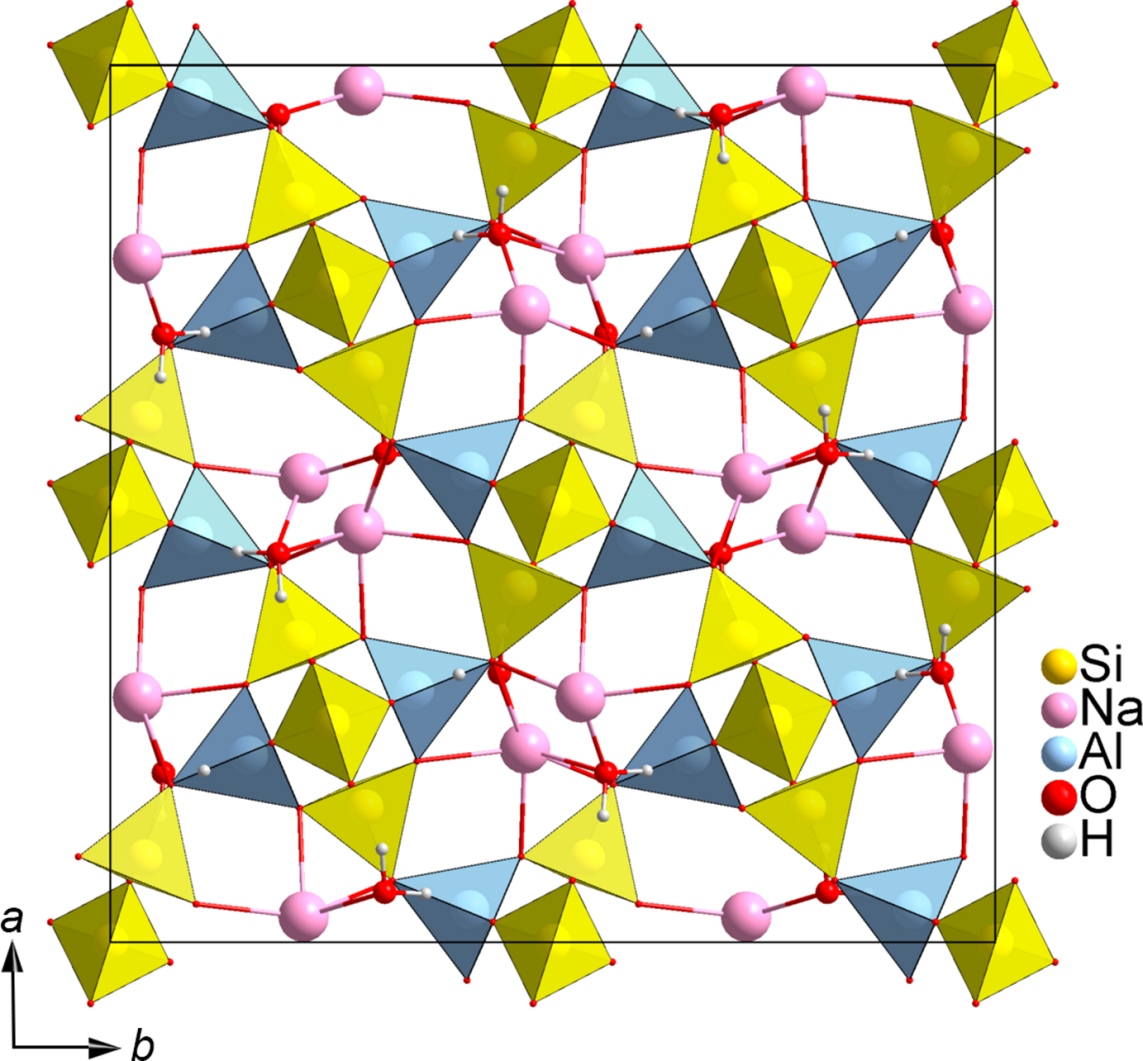
The crystal structure of natrolite along the [001] direction.

The structure was solved using the dual-space method with *SHELXT* and least-squares refinement was performed using *SHELXL* (Sheldrick, 2015*b* ▸). All atoms were refined anisotropically. The extinction parameter EXTI was applied in the refinement. This was required to partially compensate for the dynamical effects on the intensities, thereby avoiding anisotropic displacement parameters for some atoms from becoming non-positive definite (NPD), and to stabilize the positions of H atoms, which were not fixed. The structure is shown in Fig. 3 ▸.

#### Investigation of reindexing possibilities

Natrolite is pseudo-tetra­gonal with *a* ≈ *b*. Errors on ob­ser­ved unit-cell dimensions are generally higher from 3D ED data on a standard Transmission Electron Microscope (TEM) than from an X-ray dif­frac­tom­eter. We considered the possibility that individual data sets may have been misindexed, swapping the *a* and *b* axes. In many cases such a situation would be associated with poor merging statistics. Programs such as *dials.symmetry* allow automatic reindexing of data sets to form a consistent set. However, for natrolite, the effect of misindexing on the diffraction intensities is subtle. Indeed, the pro­gram combines the three data sets assuming tetra­gonal symmetry. Scaling under these constraints produced merging statistics that are similar [overall *R*_meas_(*I*) = 0.235 compared to 0.198 from the run in *Fdd*2]. Subsequent phasing by *SHELXT* gave a solution in the space group *I*
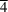
2*d*, which is the space group for gonnardite, a related mineral with a disordered natrolite framework (Artioli & Galli, 1999 ▸). We could not use *dials.symmetry* to automatically determine the correct reindexing possibilities for ortho­rhom­bic natrolite; however, with just three data sets to consider, and without treating one data set as a reference, there are only eight possible combinations in which between zero and three data sets are reindexed to swap the similar length axes. For any particular data set, that was achieved by replacing the *dials.reindex* command in the listing in Section 2.2 with the following command, and otherwise processing identically:

dials.reindex indexed.expt indexed.refl\

change_of_basis_op=c,b,-a space_group=Fdd2

For all eight runs, *SHELXT* found a solution in *Fdd*2. An initial refinement of the solutions showed clearly better results for the run in which no reindexing was performed, therefore the initial assignment of axes was found to be correct. Refinement results are presented in Table S4.

## Rigaku Synergy-ED data

Data sets were collected at the UK National Electron Diffraction Facility (NEDF) site at the University of Southampton. The facility is equipped with Rigaku XtaLAB Synergy-ED instruments, combining a JEOL JSM-2300ED electron source optimized for diffraction studies at 200 kV, with a Rigaku HyPix-ED hybrid pixel array detector. We chose nine data sets of l-histidine chloride monohydrate collected at a temperature of 175 K by NEDF staff as our example of processing with *DIALS*. Two data sets were collected with a tilt per image of 0.25°, while the remaining five used 0.2° per image. Total tilt ranged from 112 to 126.4°.

### Data processing

The *DIALS* commands used to integrate one of the histidine data sets are as follows:

dials.import\

"$HISTIDINE_DATA"/exp_705/frames/*.rodhypix\

panel.gain=2.9\

geometry.goniometer.axes=0,-1,0,\

0,-0.642788,0.766044,\

0.050593,-0.99872,0\

image_range=1,484

dials.find_spots imported.expt d_max=10 gain=0.5

dials.index imported.expt strong.refl\

detector.fix=distance space_group=P212121\

max_lattices=2 minimum_angular_separation=1

dials.refine indexed.expt indexed.refl\

detector.fix=distance

dials.integrate refined.expt refined.refl\

prediction.d_min=0.6

The path to the parent directory containing the histidine data sets is given by the variable HISTIDINE_DATA.

#### Importing the data set

The Rigaku Oxford Diffraction file format contains a comprehensive specification of diffraction geometry as metadata. The *dxtbx* format class used to read these images does not read all metadata items, but extracts enough from the header to create a model for the experiment. The gain is assumed to be equal to 2.9 and is set at import. This value was determined experimentally using low dose imaging to identify single electron impacts (Robert Bücker, personal communication). As with the natrolite example, we found that the sensitivity of spot-finding was too low using this gain value, so we passed the option gain=0.5 to *dials.find_spots* to ensure weak spots were located.

One complication with images from the Rigaku dif­frac­tom­eter is that the incremental image number that forms part of the filename is not zero-padded. For example, the 496 images of the exp_705 data set begin with file exp_705_1_1.rodhypix and end with exp_705_1_496.rodhypix. This is counter to the typical scheme used at synchrotron beamlines and other facilities, in which the final number has a fixed width, such as 0001 to 0496. Initially *DIALS* could not inter­pret this image filename template as a contiguous range of images, and we had to rename images to conform to a zero-padded template. During the course of this work, we added the ability to read filename templates with non-zero-padded image numbers.

The orientation of the rotation axis is read from the image metadata, which in this case locates it exactly anti­parallel to the *y* axis. In fact, runs of *dials.find_rotation_axis* on each data set gave a corrected axis orientation about 2.9° from this nominal orientation. We found that it was not necessary to correct the orientation in order to successfully index each of the example data sets; however, in general, it is best to determine the rotation axis using good data sets and then set it to process all other data sets where it should be the same. The Rigaku Oxford Diffraction format contains a description for a multi-axis goniometer, even though for the Synergy-ED instrument only a single axis is used. In order to set the corrected axis at import, we used the geometry.goniometer.axes option to set nine values, three for each axis in order from the sample to the goniometer base. These values were taken from the *dials.find_rotation_axis* log file for one of the runs.

These data sets contain some images at the extremes of the tilt range where part of the diffraction pattern is shadowed by the sample holder. We identified these images by inspection using the *dials.image_viewer* and then chose to exclude them from import using the image_range parameter.

#### Indexing multiple lattices

During the initial processing of these data sets, we found that the percentage of indexed spots was generally high, though somewhat lower for three of the data sets: exp_705, exp_710 and exp_712. Explorations with *dials.reciprocal_lattice_viewer* revealed the presence of more than one lattice in these cases. We indexed the second lattice for exp_705 by providing the option max_lattices=2 to *dials.index*, as shown in the example processing commands. This option causes the pro­gram to attempt to index again from the unindexed spots remaining after the first lattice is found. When an additional lattice is found, *dials.index* will reject it if it is in too similar an orientation to any previously-accepted lattice, under the control of the minimum_angular_separation parameter. The default value for this is 5°, which for the example data set exp_705 resulted in rejection of the second lattice, which was rotated by just 3.3° from the first. Reducing the value to minimum_angular_separation=1 en­sured that the second lattice was retained. Single lattice indexing assigned 59% of the found spots to one crystal model, while two lattice indexing assigned 52% of spots to the first lattice and a further 42% of spots to the second lattice. The decrease in proportion of spots assigned to the first lattice indicates the reassignment of spots that are more appropriately indexed by the second lattice. Finding the second lattice and reassigning those spots therefore improves the indexing results for the first lattice too.

Processing both lattices from exp_705 improved the overall merged data set, whereas conversely the additional lattices found in exp_710 and exp_712 diffracted weakly and reduced the overall quality of merged data. Therefore, we integrated only the major lattice in these two cases.

When there are multiple lattices in a single data set, it is likely that some diffraction spots will overlap, adding error to integrated intensities. This is particularly the case for lower resolution reflections of closely-aligned crystals. If all lattices that are present are successfully indexed, the information is available in principle to detect the spots that overlap and either reject their intensities, or attempt to deconvolute them. Unfortunately, *dials.integrate* does not offer this facility yet. We investigated overlapped reflections in this case and found that they were also not removed as outliers by *dials.scale* (see supplementary Section S2). Nevertheless, we encountered no difficulties in solving and refining the structure, so may conclude that errors from overlaps in exp_705 are tolerable in this case.

#### Combining data sets, scaling and export

An overall unit cell was refined against all of the integrated data sets with the following command:

dials.two_theta_refine\

"$HISTIDINE_PROC"/exp_705/integrated.{expt,refl}\

"$HISTIDINE_PROC"/exp_706/integrated.{expt,refl}\

"$HISTIDINE_PROC"/exp_707/integrated.{expt,refl}\

"$HISTIDINE_PROC"/exp_708/integrated.{expt,refl}\

"$HISTIDINE_PROC"/exp_710/integrated.{expt,refl}\

"$HISTIDINE_PROC"/exp_711/integrated.{expt,refl}\

"$HISTIDINE_PROC"/exp_712/integrated.{expt,refl}\

"$HISTIDINE_PROC"/exp_713/integrated.{expt,refl}\

"$HISTIDINE_PROC"/exp_715/integrated.{expt,refl}

Here we assume that the processing of each data set was performed in its own directory, under a common parent directory, and we have provided the path to that parent directory in the HISTIDINE_PROC variable. The unit cell with standard uncertainties was determined to be *a* = 6.7936 (3), *b* = 8.8294 (4) and *c* = 15.1621 (10) Å, with the space group *P*2_1_2_1_2_1_. This unit cell was carried through to the exported *SHELX* .ins file and later used for model refinement. The commands used to scale and export the data were:

dials.scale\

refined_cell.expt\

"$HISTIDINE_PROC"/exp_705/integrated.refl\

"$HISTIDINE_PROC"/exp_706/integrated.refl\

"$HISTIDINE_PROC"/exp_707/integrated.refl\

"$HISTIDINE_PROC"/exp_708/integrated.refl\

"$HISTIDINE_PROC"/exp_710/integrated.refl\

"$HISTIDINE_PROC"/exp_711/integrated.refl\

"$HISTIDINE_PROC"/exp_712/integrated.refl\

"$HISTIDINE_PROC"/exp_713/integrated.refl\

"$HISTIDINE_PROC"/exp_715/integrated.refl\

merging.nbins=10\

d_min=0.64

dials.export scaled.expt scaled.refl\

format=shelx composition="C H N O Cl"

Merging statistics from the *dials.scale* run with options as above are given in Table 2 ▸.

### Structure solution and refinement

**Figure 4:**
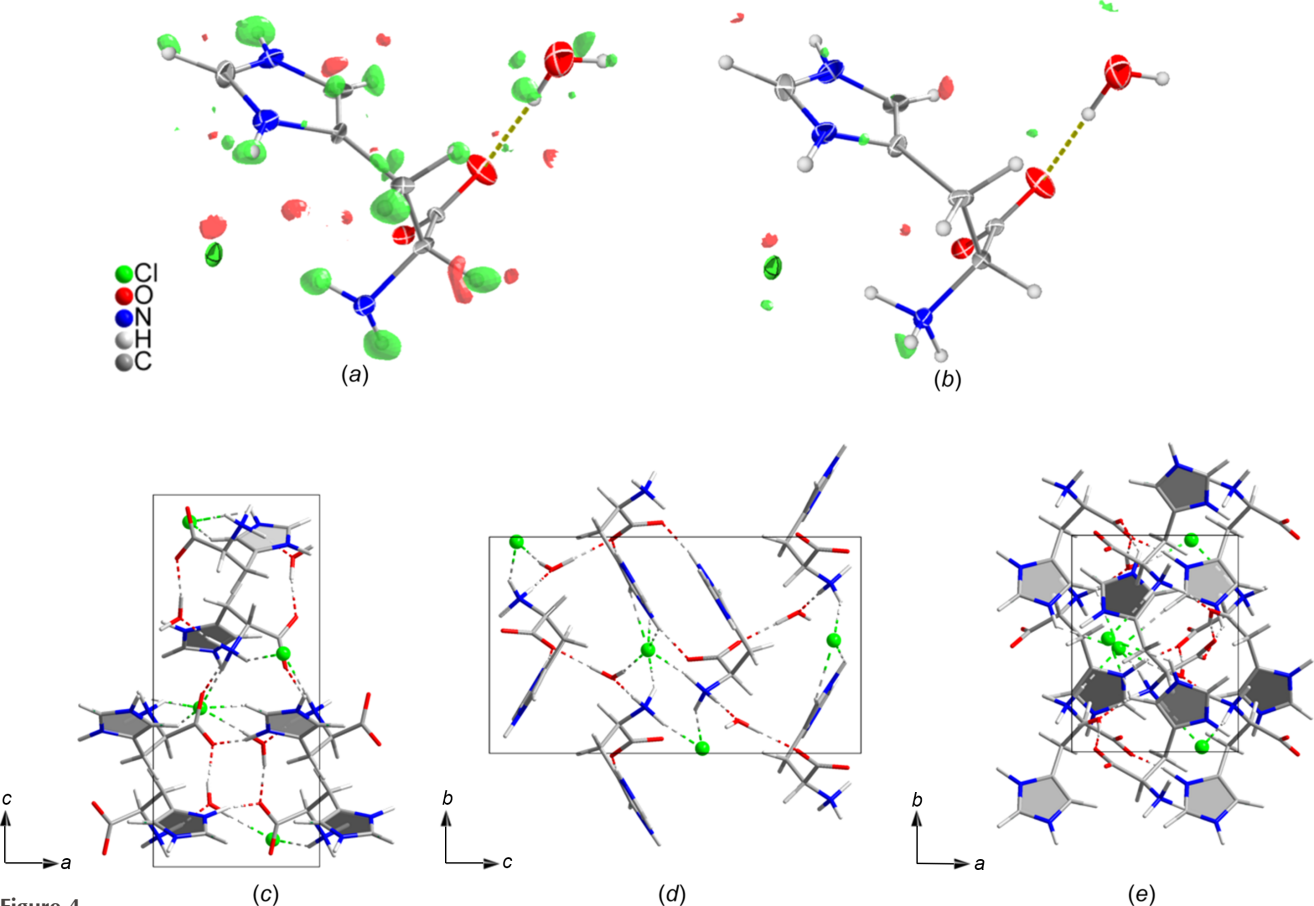
The structural model of l-histidine. An asymmetric unit superimposed with the *F*_obs_–*F*_calc_ map contoured at 3σ calculated (*a*) before any H atoms were added and (*b*) before adding the last H atom. H-atom positions were placed where they were expected to be based on the positive density peaks from the *F*_obs_–*F*_calc_ omit map. Positive peaks are shown in green and negative ones are shown in red. The structure model viewed along the (*c*) [100], (*d*) [010] and (*e*) [001] directions, showing the hydrogen-bond network.

The structure was solved by the dual-space method using *SHELXT* and least-squares refinement was performed using *SHELXL*. All non-H atoms were refined anisotropically. The extinction parameter EXTI was applied in the refinement to stabilize the refinement of H-atom positions. It was possible to locate all H atoms during kinematical refinement from the Fourier difference map, as shown in Fig. 4 ▸. The last H atom was added after a refinement run after adding all other H atoms. Then H-atom ADPs were refined one atom at a time. Since the density peak for the last H atom was on the noise level and the N—H bond distance appeared to be very long (1.2 Å), the distance between these atoms was restrained. Table 2 ▸ summarizes the crystallographic data and refinement results and the structure is shown in Fig. 4 ▸.

#### Determining the absolute hand

Data for the best diffracting histidine crystal, exp_715, were exported as ‘virtual frames’ (Klar *et al.*, 2023 ▸) by adopting the format pioneered by *PETS2*. The *DIALS* command used to do this was:

<!?h 16pt>dials.export\

"$HISTIDINE_PROC"/exp_715/integrated.{expt,refl}\

format=pets n_merged=5 step=3

The n_merged parameter controls the number of real frames to merge in a virtual frame, while step selects the number of frames between each virtual frame. The step size is smaller than the number of frames to merge, so that virtual frames overlap. The output of this command is the file dials_dyn.cif_pets that can be passed to *JANA2020* for dynamical refinement, as detailed in Klar *et al.* (2023 ▸). By this pro­cedure, the correct l-enanti­omer was determined suc­cess­fully as the one giving the smaller *R* value, with confidence as reported by the *z*-score, as calculated by *jana_tools* (Klar, 2023 ▸). These dynamical refinement results are summarized in Table 2 ▸.

## Eldico ED-1 data

The Eldico ED-1 instrument is a dedicated electron dif­frac­tom­eter, with a high-precision goniometer and a horizontal layout, minimizing the sphere of confusion at the sample position (Simoncic *et al.*, 2023 ▸; Heidler *et al.*, 2019 ▸). The dif­frac­tom­eter has a 160 kV LaB_6_ electron source and no post-sample lenses. This simple yet robust design avoids distortions in diffraction patterns that are otherwise common with 3D ED data (Brázda *et al.*, 2022 ▸), enabling accurate unit-cell determination. The trade-off is that it is not possible to alter the effective detector distance. For the current instrument, this means it is not possible to analyse samples with very large unit cells. For example, if a minimum spacing of four pixels between spot centres is required for successful data processing of a particular crystal, then at the detector distance of 578.3 mm used for the data sets we investigated, the maximum unit-cell dimension would be 55 Å. For crystals where spot size is increased due to factors such as high mosaicity, the minimum spacing between resolved spots may be larger, further limiting the maximum unit-cell dimension. Nevertheless, in principle, the design still permits adjustments of the mechanical detector distance, exactly as with an X-ray beamline.

For illustration of data processing, four data sets from crystals of 1,3,5-tri­phenyl­benzene (TPB), collected at room temperature by Eldico staff, were chosen. Sample preparation and data collection details were the same as published previously in Simoncic *et al.* (2023 ▸). Each data set was collected with a tilt per image of 0.5° and total tilt ranged from 85 to 110°.

### Data processing

The *DIALS* commands used to integrate each data set were equivalent. Here we show the script for one of the data sets, where the parent directory containing each data set directory is given by the variable TPB_DATA:

dials.import "$TPB_DATA"/03/*.cbf\

panel.gain=1.6\

geometry.goniometer.axis=-0.052336,0.99863,0

dials.find_spots imported.expt d_max=10

dials.index imported.expt strong.refl\

detector.fix=distance space_group=P222

dials.refine indexed.expt indexed.refl\

detector.fix=distance\

crystal.unit_cell.force_static=True

dials.integrate refined.expt refined.refl\

prediction.d_min=0.7

#### Importing the data set

Data from the ED-1 instrument was provided in miniCBF format, which is a relatively simple file format suitable for capturing diffraction geometry information from conventional rotation experiments with a single axis orthogonal to the beam direction. *DIALS* reads miniCBF format natively; nevertheless, we added a format class specific for the ED-1 instrument to the *dxtbx* library. This allowed us to set some specific defaults, ensuring an unpolarized electron beam model was created, and that the parallax and QE (Quantum Efficiency) corrections controlled by the detector model were disabled, as the form of these corrections in *DIALS* is appropriate only for X-ray data. The *DIALS* inter­pretation of these files places the rotation axis exactly along the *y* axis. In fact, runs of *dials.find_rotation_axis* indicated that the true axis is about 3° offset from this. As the rotation axis is fixed and there are no post-sample lenses, this value can be considered a constant for the instrument. As for previous examples, we set the orientation of the axis explicitly as an option to the *dials.import* command.

The miniCBF images do not contain metadata giving the detector gain. We performed a similar analysis to that presented in Section 2.2.2 to estimate the gain to be 1.6. In contrast to the previous two examples, we found that spot-finding did not require an increase in sensitivity to capture weak spots. In fact, setting gain=0.5 as an option to *dials.find_spots* in this case resulted in the algorithm being too sensitive, with many noise spots being found in addition to the Bragg peaks.

#### Further processing

The space group of TPB is *Pna*2_1_, but it is unnecessary to set the exact space group for integration. What matters is that the Bravais lattice is correct. Here we ensured that by using the subgroup *P*222 to enforce ortho­rhom­bic lattice constraints, then allowed the correct space group to be found during structure solution by *SHELXT*.

The unit cells of the four indexed data sets were highly consistent, shown in Table S3. This may be attributed to the lack of post-sample lenses and the precision of the goniometer of the ED-1 instrumental. We found that scan-varying refinement of the unit cells showed only small changes during each scan, with the greatest change being an increase of 0.1 Å in the *c* parameter of data set 07. Merging statistics were no better using a scan-varying model for the cell as compared with allowing only the crystal orientation to vary smoothly with scan position. Therefore, we continued with this simpler model for each data set, by setting the option crystal.unit_cell.force_static=True for *dials.refine.*

#### Combining data sets, scaling and export

The integrated data sets were combined and a single unit cell was refined using this *dials.two_theta_refine* command, where the path to the parent directory containing processed data sets was stored in the variable TPB_PROC:

dials.two_theta_refine\

"$TPB_PROC"/03/integrated.{expt,refl}\

"$TPB_PROC"/06/integrated.{expt,refl}\

"$TPB_PROC"/07/integrated.{expt,refl}\

"$TPB_PROC"/08/integrated.{expt,refl}

The unit cell determined by this procedure was *a* = 7.5894 (4), *b* = 11.2305 (4) and *c* = 19.7150 (6) Å. Joint scaling and export of the four TPB data sets was achieved with these commands:

dials.scale refined_cell.expt\

"$TPB_PROC"/03/integrated.refl\

"$TPB_PROC"/06/integrated.refl\

"$TPB_PROC"/07/integrated.refl\

"$TPB_PROC"/08/integrated.refl\

d_min=0.75

dials.export scaled.expt scaled.refl\

format=shelx composition="C H"

We explored the use of Δ*CC*_1/2_ filtering but found no advantage for these data sets. Merging statistics for this *dials.scale* run are given in Table 3 ▸.

### Structure solution and refinement

**Figure 5:**
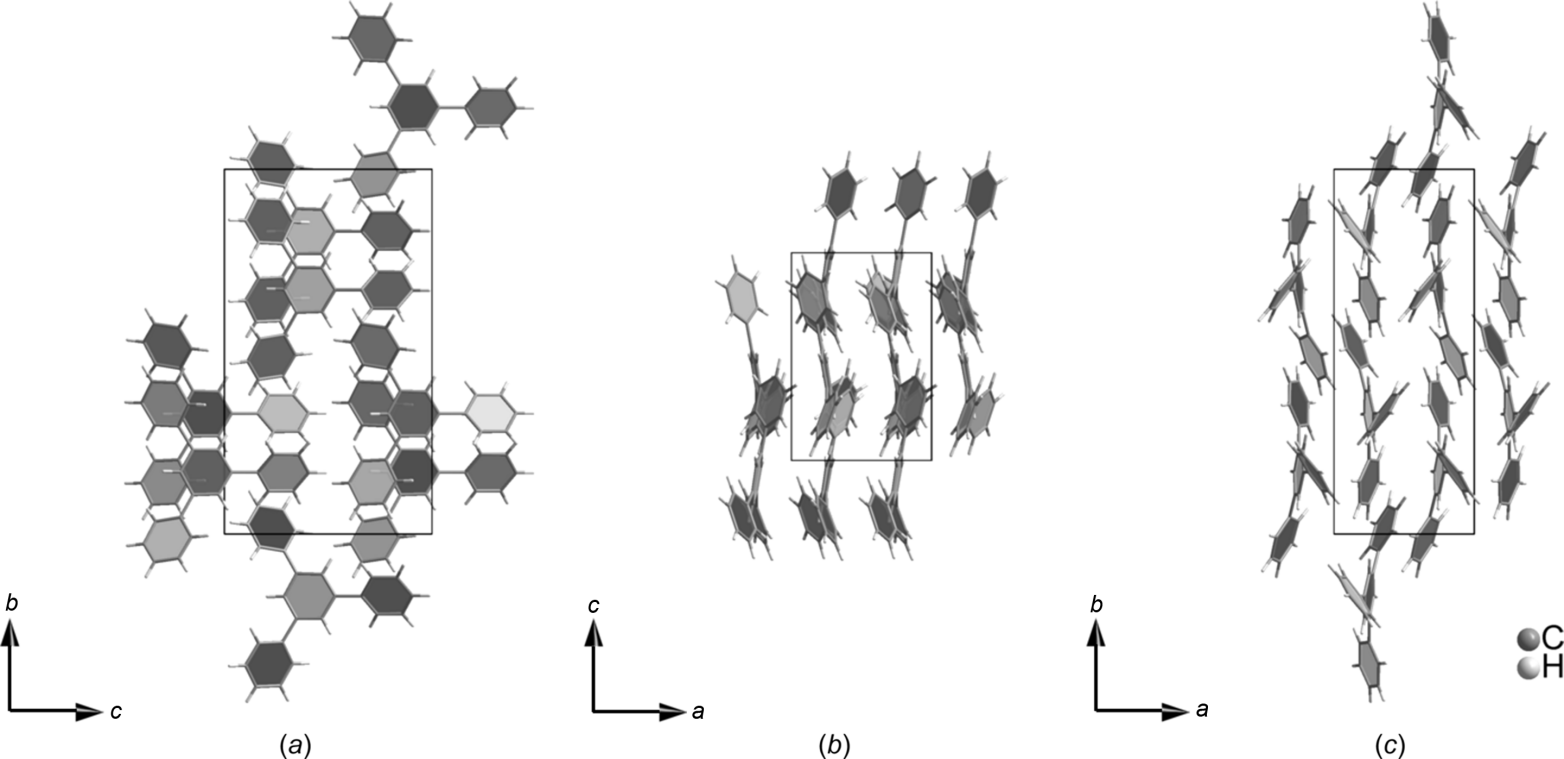
Projection of the TPB unit cell along the (*a*) [100], (*b*) [010] and (*c*) [001] directions.

Structure solution was performed, as with the natrolite and histidine examples, with *SHELXT*. Refinement was made using *SHELXL*. All non-H atoms were refined anisotropically with no restraints. H atoms were added in riding positions, and the extinction parameter EXTI was applied in the refinement. The structure is shown in Fig. 5 ▸.

## Conclusions

The development of 3D ED has enhanced structure determination in chemical crystallography, enabling nano-crystallography from samples that previously could only be in­vestigated by bulk *via* powder diffraction (Huang *et al.*, 2021 ▸; Gruene & Mugnaioli, 2021 ▸; Gemmi *et al.*, 2019 ▸). This technique has benefited over recent years from optimizations in instrumentation, data collection protocols and processing. In particular, the use of pixel array detectors for 3D ED applications has enabled rapid data collection free from readout noise. Fast data collection in turn facilitates the fine-sliced con­tin­u­ous rotation methodology that is the standard in X-ray crystallography. Data processing software used in X-ray crystallography is now a popular choice, as discounting the effect of post sample lenses, the effective diffraction geometry of the con­tin­u­ous rotation experiment is equivalent. Thus, the advances in data processing that improved data quality in X-ray crystallography, such as 3D profile fitting, may be applied to 3D ED data too.

*DIALS* is a popular package for single crystal data pro­cessing, under current active development. Recent additions to the *DIALS* package have improved its usability for 3D ED, so that it now provides a unified easily-scriptable framework for data processing across X-ray and electron diffraction. We demonstrate the use of *DIALS* for 3D ED of small mol­ecule and framework structures with three examples from different instruments, with the common factor that data were recorded with a high-quality pixel array detector in each case. The first example shows how the software was adapted for data from an academic lab with a bespoke setup, in which regular calibration images were taken, inter­rupting the con­tin­u­ous sweep of diffraction data. The second and third examples come from commercial instruments, which we may expect to become increasingly important as the field matures. High quality data is achievable in each case. For the chiral amino acid l-histidine, we demonstrate how data processed with *DIALS* may be used for determining the absolute structure by emulating the output format used by *PETS2*, which is readable by *JANA2020* for dynamic diffraction-based refinement.

Future developments in *DIALS* for 3D ED should address some remaining deficiencies. For the Timepix and HyPix-ED detectors with a higher event multiplicity, or gain, the default spot-finding algorithm was found to be insensitive to weak spots. The gain was effectively halved for this step to increase sensitivity. Improvements to the spot-finding algorithm may be sought to make this adjustment unnecessary. The precise determination of unit-cell parameters is best demonstrated with the TPB data, where the lack of post-sample lenses on the Eldico ED-1 diffraction means there are no distortions introduced into the diffraction pattern. Comprehensive modelling of distortions has already been demonstrated (Brázda *et al.*, 2022 ▸), and this provides another obvious direction for improvements in *DIALS* for data collected on a TEM. Our experiences indicate that the standard method of error modelling developed for X-ray data typically underestimates the true error. Existing work to address this fact (Khouchen *et al.*, 2023 ▸) should inform developments in *DIALS*. We also found that the symmetry-determination tools in *DIALS* that can be used to resolve indexing ambiguities were not as effective with 3D ED data as they are with X-ray data, and their use is not demonstrated here. Work to improve the robustness of these tools will be important to simplify the workflow in cases in which the correct space group is not previously known.

*DIALS* is an open-source community project. The growing community of practitioners performing 3D ED includes methods developers, and we would like to make it clear that contributions from software developers inter­ested in 3D ED are very welcome.

## Supplementary Material

Crystal structure: contains datablock(s) natrolite, histidine, tpb, global. DOI: 10.1107/S2053229624011148/yp3238sup1.cif

Additional text, tables and figures. DOI: 10.1107/S2053229624011148/yp3238sup2.pdf

CCDC references: 2403195, 2403194, 2403193

## Figures and Tables

**Table 1:** Statistics for joint scaling of three natrolite data sets

**Crystallographic data and merging statistics**			
Mol­ecular formula	Na_2_Al_2_Si_3_O_10_·2H_2_O		
Space group	*Fdd*2		
*a*, *b*, *c* (Å)	18.640 (9), 18.788 (4), 6.8419 (16)		
	Overall	Low	High
High resolution limit	0.61	1.31	0.61
Low resolution limit	6.62	6.62	0.63
Completeness (%)	95.7	100	71.1
Multiplicity	7.2	8.3	3.2
*I*/σ	13.0	39.3	2.2
*R*_meas_(*I*)	0.198	0.115	0.515
*R*_pim_(*I*)	0.067	0.039	0.261
*CC*_1/2_	0.991	0.994	0.474
Total observations	10211	1395	345
Total unique	1410	168	108
			
**Kinematical refinement**			
Reflections		All	[*I* > 2σ(*I*)]
Independent reflections		2469	1948
*R*_1_		0.1681	0.1448
*wR*_2_		0.3767	0.3557
Goof		1.175	

**Table 2:** Statistics for joint scaling of nine histidine data sets

**Crystallographic data and merging statistics**			
Mol­ecular formula	C_6_H_10_ClN_3_O_2_		
Space group	*P*2_1_2_1_2_1_		
*a*, *b*, *c* (Å)	6.795 (2), 8.824 (2), 15.149 (3)		
	Overall	Low	High
High resolution limit	0.64	1.38	0.64
Low resolution limit	8.83	8.83	0.66
Completeness (%)	100	100	100
Multiplicity	40.4	35.9	35.6
*I*/σ	13.9	53.0	1.8
*R*_meas_(*I*)	0.430	0.231	1.946
*R*_pim_(*I*)	0.065	0.037	0.333
*CC*_1/2_	0.997	0.997	0.350
Total observations	85372	8826	7296
Total unique	2115	246	205
			
**Kinematical refinement**			
Reflections		All	[*I* > 2σ(*I*)]
Independent reflections		3641	2589
*R*_1_		0.1484	0.1231
*wR*_2_		0.3237	0.3132
Goof		1.126	
			
**Dynamical refinement**			
		L-His	D-His
*R*_1_(all)		0.1904	0.2117
*R*_1_(obs)		0.1269	0.1473
*wR*_2_(all)		0.2677	0.2997
*wR*_2_(obs)		0.2380	0.2696
*z*-score		5.1σ	

**Table 3:** Statistics for joint scaling of four TPB data sets

**Crystallographic data and merging statistics**			
Mol­ecular formula	C_24_H_18_		
Space group	*Pna*2_1_		
*a*, *b*, *c* (Å)	7.5894 (4), 11.2305 (4), 19.7150 (6)		
	Overall	Low	High
High resolution limit	0.75	1.61	0.75
Low resolution limit	9.86	9.86	0.78
Completeness (%)	99.3	98.6	96.8
Multiplicity	12.5	12.2	6.8
*I*/σ	5.5	20.9	0.4
*R*_meas_(*I*)	0.230	0.140	1.541
*R*_pim_(*I*)	0.061	0.039	0.564
*CC*_1/2_	0.993	0.991	0.295
Total observations	29950	3388	1465
Total unique	2391	277	214
			
**Kinematical refinement**			
Reflections		All	[*I* > 2σ(*I*)]
Independent reflections		4137	1778
*R*_1_		0.1685	0.1087
*wR*_2_		0.2796	0.2511
Goof		0.889	

## Data Availability

The data sets described in this article are available to download on Zenodo. The direct links are: Natrolite, https://zenodo.org/records/12592764; Histidine, https://zenodo.org/records/10974780; TPB, https://zenodo.org/records/11119252. Data processing scripts for the examples presented here are made available on GitHub at https://github.com/aimeon/DIALS-proc. For the purpose of open access, the author has applied a Creative Commons Attribution (CC BY) licence to any Author Accepted Manuscript version arising.
