# Supplementary material for: How to use DIALS to process chemical crystallography 3D ED rotation data from pixel array detectors
Source: Acta Crystallogr C Struct Chem. 2025 Jan 1;81(Pt 1):1–13. doi: 10.1107/S2053229624011148 (PMC11700371; doi:10.1107/S2053229624011148)
Supplement: Supplementary file 2 [file c-81-00001-sup2.pdf]

STRUCTURAL CHEMISTRY

Volume 80 (2024)

Supporting information for article:

How to use *DIALS* to process chemical crystallography 3D ED rotation data from pixel array detectors

Angelina Vypritskaia, Xiaodong Zou, Taimin Yang and David Geoffrey Waterman

S1. Gain determination from low dose images

Low dose images were collected on the Timepix detector as described in Section 2.2.2, and an example of this image data is shown in Figure S1. 40 images were collected and analysed by the script *count_event_multiplicity.py*, available from https://github.com/aimeon/count_event_multiplicity. In total, the multiplicity of 4868 clusters was averaged, giving the value 2.9 ± 0.01.

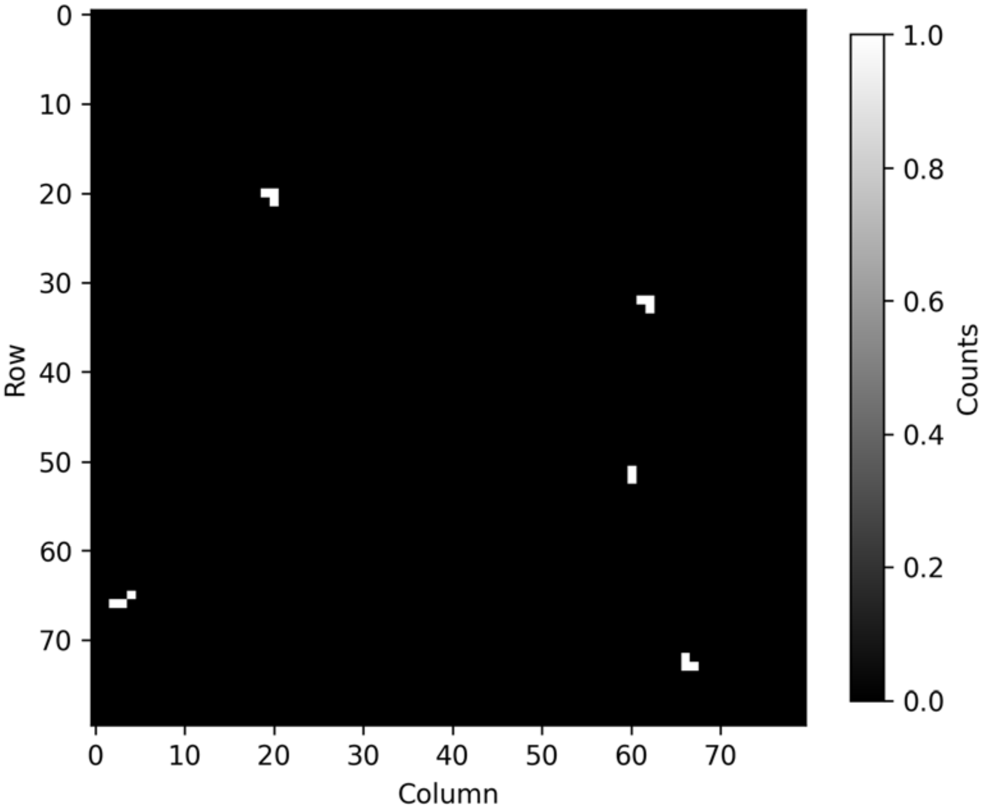

Figure S1 A region of interest of 80×80 pixels from one of the low dose images collected on the Timepix detector. Clusters are assumed to result from single 200 keV electron impacts.

Table S1 Indexing results for three natrolite data sets with *F*222 symmetry imposed.

Data set	Number of strong spots		RMSD (pixels)			Unit cell dimensions (Å)		
	Indexed	Unindexed	*X*	*Y*	*Z*	*a*	*b*	*c*
Data1	3219	582	1.38	1.06	0.84	6.8356(10)	18.867(4)	18.849(3)
Data3	2475	2010	0.92	1.12	1.50	6.7272(13)	18.136(5)	18.624(5)
Data4	2805	139	0.68	1.08	0.59	6.8451(8)	18.543(3)	18.737(3)

In the case of Data3 spots were split, leading to many unindexed spots forming a second lattice. However, indexing the second lattice did not improve data processing, so the unindexed spots were ignored.

Table S2 Indexing results for nine histidine data sets in space group $P2_12_12_1$.

Data set	Number of strong spots		RMSD (pixels)			Unit cell dimensions (Å)		
	Indexed	Unindexed	*X*	*Y*	*Z*	*a*	*b*	*c*
exp_705	1683	125	0.23	0.20	0.24	6.7851(4)	8.8213(7)	15.1687(12)
	1348		0.25	0.21	0.34	6.7847(5)	8.8247(7)	15.1676(13)
exp_706	846	2	0.22	0.19	0.21	6.7970(7)	8.8160(9)	15.1923(15)
exp_707	402	122	0.24	0.24	0.79	6.789(3)	8.830(4)	15.194(6)
exp_708	1087	324	0.18	0.21	0.85	6.7890(15)	8.839(2)	15.200(3)
exp_710	1113	455	0.22	0.24	0.33	6.7802(6)	8.8838(11)	15.211(2)
exp_711	435	54	0.17	0.18	0.29	6.79394(10)	8.8189(2)	15.2053(9)
exp_712	384	286	0.23	0.28	0.81	6.787(3)	8.811(4)	15.233(8)
exp_713	1399	70	0.52	0.39	0.57	6.74083(12)	8.9072(3)	15.1805(11)
exp_715	1240	140	0.28	0.31	0.45	6.7755(3)	8.8596(5)	15.1536(19)

Two lattices were indexed in the case of exp_705.

Table S3 Indexing results for four TPB data sets in space group *P*222.

Data set	Number of strong spots		RMSD (pixels)			Unit cell dimensions (Å)		
	Indexed	Unindexed	*X*	*Y*	*Z*	*a*	*b*	*c*
03	1987	112	0.36	0.19	0.24	7.58751(6)	11.22693(18)	19.7095(7)
06	1691	151	0.31	0.21	0.25	7.57271(6)	11.22479(19)	19.7201(8)
07	3662	184	0.36	0.26	0.22	7.58649(13)	11.2262(4)	19.7110(8)
08	2606	50	0.33	0.19	0.24	7.58898(5)	11.22841(15)	19.7030(6)

Table S4 R1 values for an initial refinement by *SHELXL* for combinations of three natrolite data sets. Results are shown for each possible combination of reindexing to swap axes *a* and *b* for individual data sets.

Reindexing scheme	Strong reflections ($F_o > 4\ \sigma(F_o)$)		All reflections	
	Number of reflections	R1	Number of reflections	R1
000	1743	0.1650	2450	0.1878
001	1896	0.1791	2518	0.1954
010	1687	0.1841	2382	0.2036
011	1782	0.1881	2448	0.2066

100	1782	0.1836	2448	0.2028
101	1687	0.1912	2382	0.2112
110	1896	0.1855	2518	0.2028
111	1743	0.1750	2450	0.1979

Reindexing possibilities are described by a 3 bit pattern, where 000 indicates that no data sets were reindexed, while 111 indicates that all three data sets were reindexed.

S2. Analysis of overlapped reflections in histidine data sets

One of the nine histidine data sets, `exp_705`, contained two distinct lattices that were processed separately. We expected that some spots would be overlapped, resulting in erroneously increased intensity for those spots. Inspection of the `dials.integrate.log` file for this data set showed that the "beam divergence" parameter, σ_B, was calculated to be about 0.0095° for each lattice. At a detector distance of 644.4 mm this angle is subtended by a distance of approximately one 0.1 mm pixel. The strong pixels are located within a peak region defined by the distance $3\sigma_B$. While this calculation is performed in a local reciprocal space coordinate system, to a simple approximation we might expect overlaps to become significant when the distance between reflection centroids is less than 3 pixels. We wrote a Python script to identify pairs of reflections that are close to each other between the two lattices. An increase in intensity was observed compared to the merged intensity for reflections closer than 3-4 pixels, as shown on Figure S2.

Reflections with discrepant intensities may be detected as outliers by *dials.scale*. This analysis calculates a normalised deviation from the merged intensity for each reflection as a Z-score. The default setting of *dials.scale* is to flag reflections with a Z-score greater than 6.0 as outliers. The plot in Figure S3 shows that, despite the increase in intensity for close overlaps, there is no clear trend towards increased Z-score. Only very few reflections are rejected as outliers with the default cutoff.

The script to perform these analyses, *find_overlaps.py*, is available at https://github.com/aimeon/DIALS-proc.

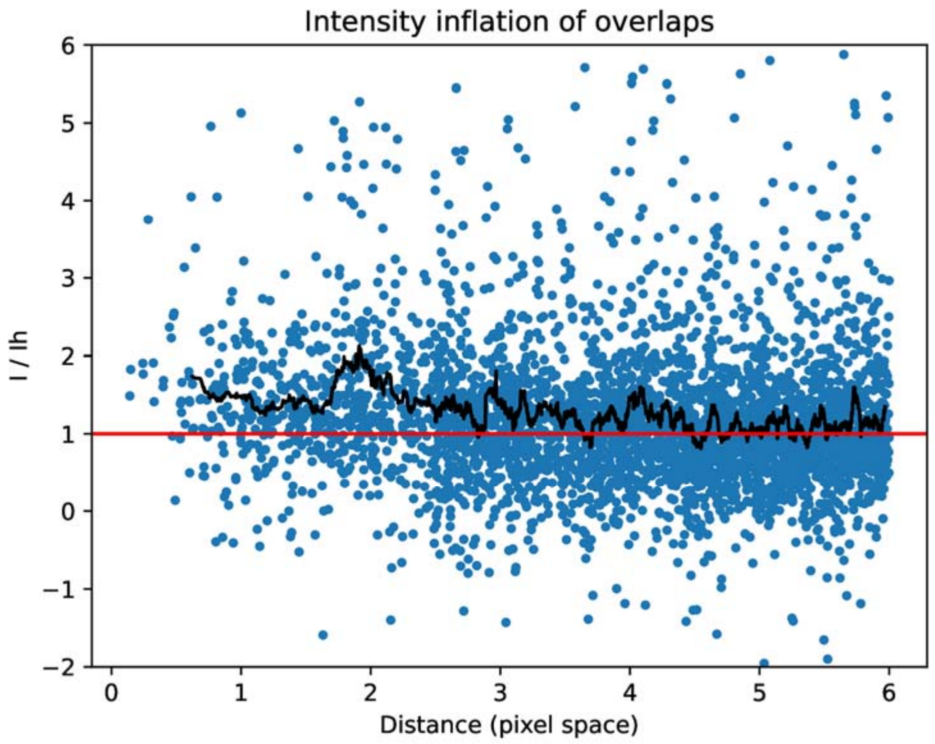

Figure S2 The ratio of individual scaled reflection intensity to the weighted average intensity of the merging group is plotted against distance between close reflections. Individual intensities are taken from the first lattice of `exp_705`, whereas merging group intensity is calculated using data from all 10 histidine lattices. A weighted average line (black) is added to help observe the trend. Reflections closer than 3-4 pixels generally have an inflated intensity compared to the merging group average.

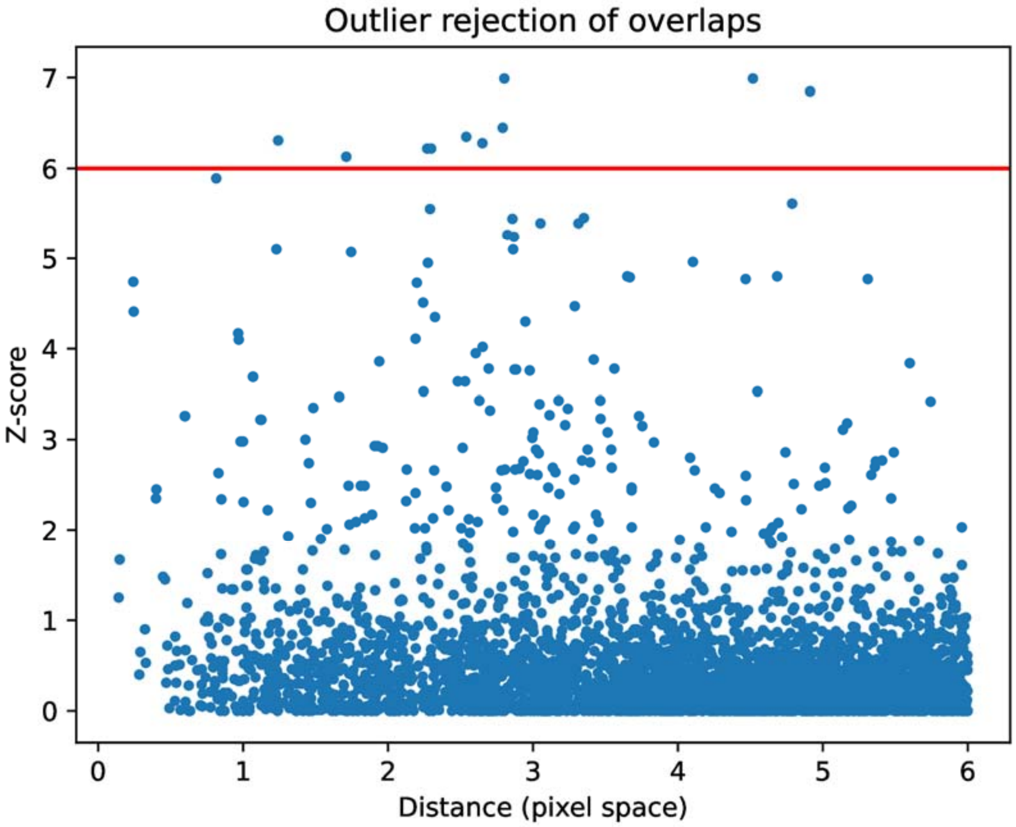

Figure S3 The outlier rejection Z-score for reflections from the first lattice of data set `exp_705` are plotted against their distance to the nearest reflection from the second lattice. Reflections with a small distance have an increased intensity due to the overlap. However, this does not result in a clear increase in Z-score. At the default cutoff for flagging as an outlier (Z=6.0, red line), only a few reflections are rejected.

S3. Scale factors for combined data sets

The program *dials.scale* produces various quality metrics in the form of tables and plots within an HTML file called dials.scale.html. A comparison of the scale factor between data sets can be seen from the plot of scale factor and R_{merge} versus image number (batch). The jointly-scaled data sets are abutted with a single monotonically increasing image number for this analysis.

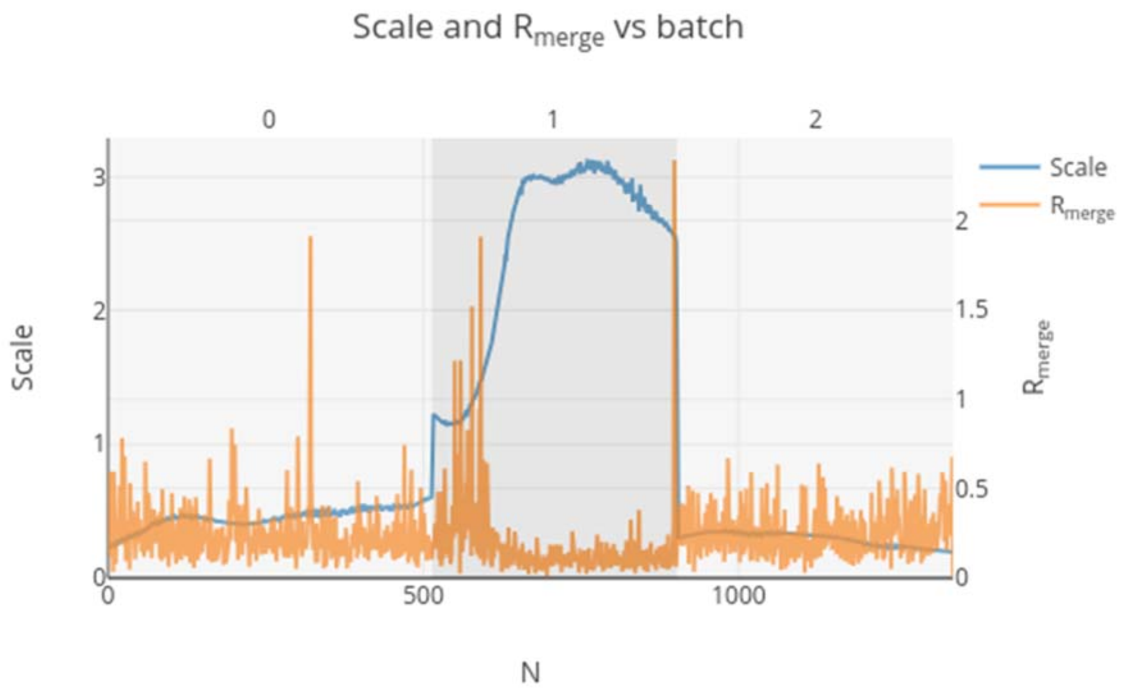

Figure S4 Scale factors and $R_{\rm merge}$ versus image number for three natrolite data sets (where background shading delineates the data sets). The scale factor here directly represents scattering strength, therefore the second data set has the strongest intensities (associated with lower $R_{\rm merge}$ values).

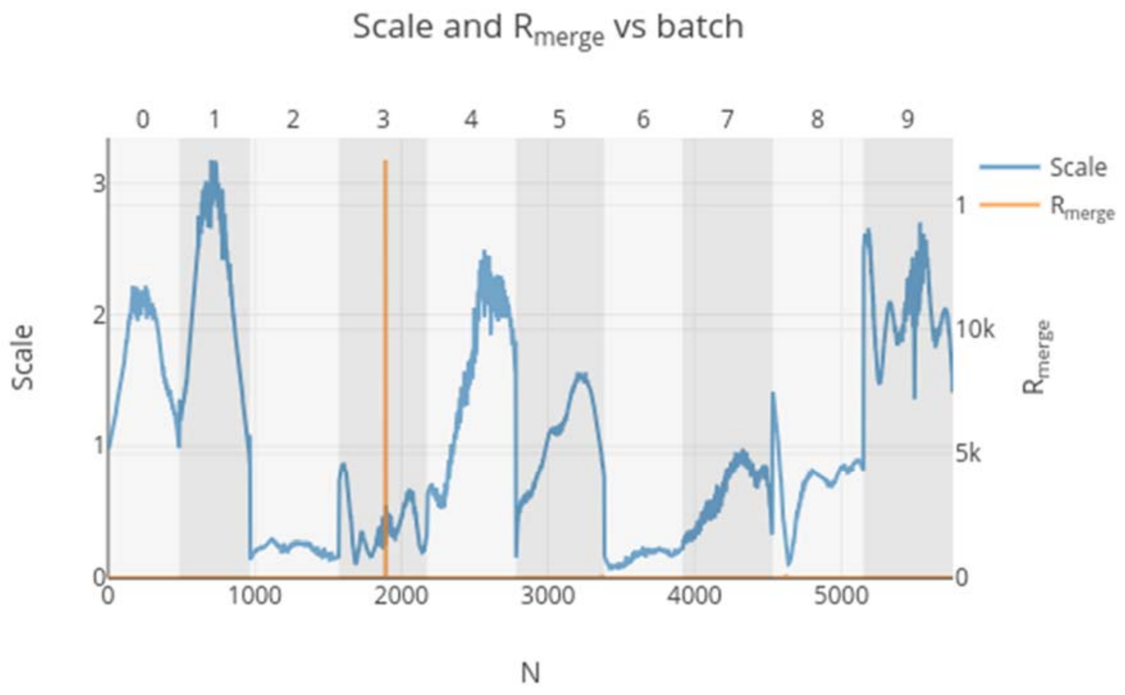

Figure S5 Scale factors and $R_{\rm merge}$ versus image number for 10 integrated lattices from histidine crystals (the first data set provided two lattices). Here a spike in $R_{\rm merge}$ is observed in the centre of the 4th data set. We have not found the cause of this spike, except that it appears to be located at a single image. Inspection of this image reveals no obvious problem. It is likely that this is due to deficiencies

in the methods used to calculate or plot the R_{merge} value rather than indicative of a real data quality issue.

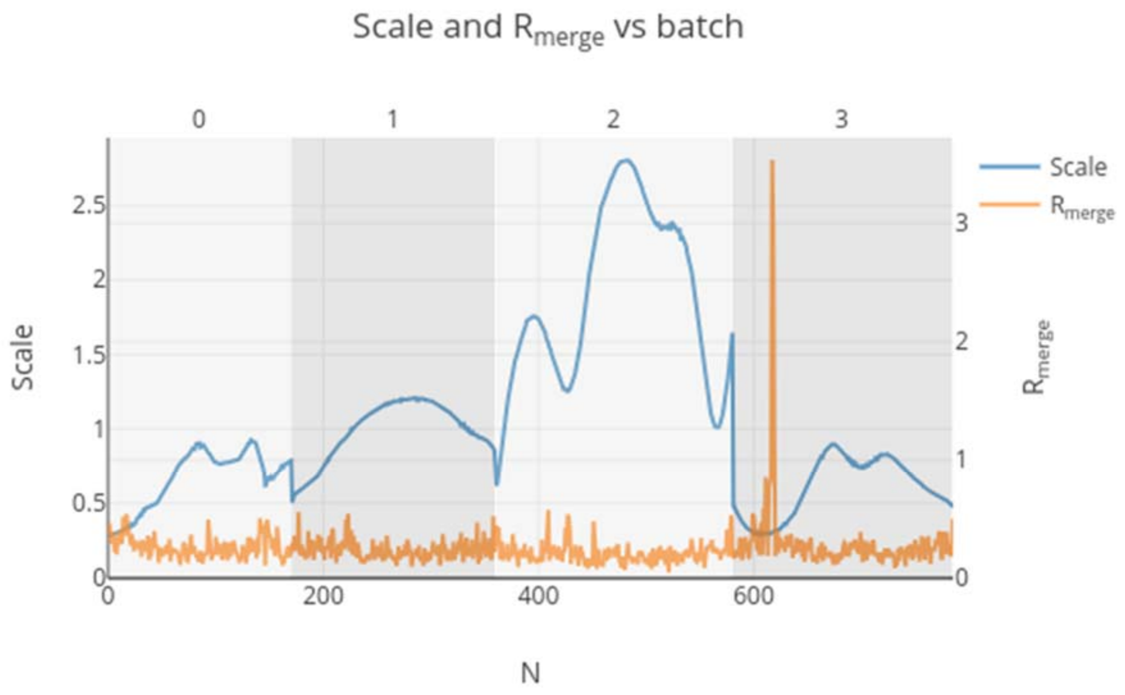

Figure S6 Scale factors and R_{merge} versus image number for four TPB data sets. A region of poor diffraction is observed at the start of the scan for the 4th crystal, with weak intensities and high R_{merge}. Nevertheless, excluding these images does not improve overall data quality.
